# ESR white paper: blockchain and medical imaging

**DOI:** 10.1186/s13244-021-01029-y

**Published:** 2021-06-22

**Authors:** European Society of Radiology (ESR), Elmar Kotter, Luis Marti-Bonmati, Adrian P. Brady, Nandita M. Desouza

**Affiliations:** Am Gestade 1, Vienna, Austria

**Keywords:** Blockchain, Radiology, Artificial intelligence, Imaging informatics, Database

## Abstract

Blockchain can be thought of as a distributed database allowing tracing of the origin of data, and who has manipulated a given data set in the past. Medical applications of blockchain technology are emerging. Blockchain has many potential applications in medical imaging, typically making use of the tracking of radiological or clinical data. Clinical applications of blockchain technology include the documentation of the contribution of different “authors” including AI algorithms to multipart reports, the documentation of the use of AI algorithms towards the diagnosis, the possibility to enhance the accessibility of relevant information in electronic medical records, and a better control of users over their personal health records. Applications of blockchain in research include a better traceability of image data within clinical trials, a better traceability of the contributions of image and annotation data for the training of AI algorithms, thus enhancing privacy and fairness, and potentially make imaging data for AI available in larger quantities. Blockchain also allows for dynamic consenting and has the potential to empower patients and giving them a better control who has accessed their health data. There are also many potential applications of blockchain technology for administrative purposes, like keeping track of learning achievements or the surveillance of medical devices. This article gives a brief introduction in the basic technology and terminology of blockchain technology and concentrates on the potential applications of blockchain in medical imaging.

## Keypoints

- Blockchain is a distributed database technology allowing to keep track of data, documenting the origin and changes of all data, including imaging data.
- Blockchain uses cryptography for integrity and authenticity of data, transparency, immutability and verifiability of data.
- Medical applications of blockchain technology are emerging, with potentially many applications in medical imaging.

## Introduction

Blockchain, a breakthrough technology, has become familiar to the public because of its widespread use in cryptocurrency markets. It has found many applications not only in industry but also in healthcare and in medical imaging [2–4], where it has been used to securely store medical data using a distributed cryptographic database where information related to the creation, update or access to medical data can be stored in a safe way [2]. Estonia has already established a complete blockchain-based healthcare ecosystem within a decade [5]. Blockchain allows users (patients, physicians, radiologists and scientists) to control how and by whom healthcare data are used. This article aims to discuss potential applications of blockchain technology in medical imaging. Challenges of these applications will be identified, and a number of use cases will be presented, including ownership and tracking of images, image annotations, and potential applications with regard to artificial intelligence. To limit the scope of this article, only a brief introduction to the basics of blockchain technology is given; for a more in-depth understanding of the technological principles, readers should refer to two recently published excellent articles [3, 4].

Blockchain can be thought of as a distributed database, tracking all changes made to the database. In contrast to classical databases, blockchain uses cryptography for integrity and authenticity of data, transparency, immutability and verifiability of data, to make information reliable through a distributed trust network, without the use of a central, trustworthy master copy (Table 1). It allows tracing of the origin of data, and who has manipulated a given data set in the past. Blockchain is potentially applicable to tracking of radiological data.

**Table 1:** Comparison of traditional database vs. blockchain (modified from McBee et al. [4])

Property	Blockchain	Traditional database
Immutability	Yes	No
Operations	Data may only be appended	Create, update, read, delete
Topology	Distributed (many nodes)	Centralized (one or few nodes)
Redundancy	Multiple due to distributed architecture	Central node is single point of failure
Consensus	Majority of peers agree on outcome of transaction	Central authority
Latency	High	Low
Transactional cost	High	Low

Blockchain technology was first described in 1991 for verifying the authenticity of digital documents via hash functions [6], and terms “block” and “chain” was first used in a paper by a person or group of persons with the pseudonym “Satoshi Nakamoto” (whose identity remains unknown). Whilst the paper uses the words block and chain, it does not frame the term blockchain. This term emerged later in a more informal setting. It laid the base for the cryptocurrency “bitcoin” following the 2007 financial crisis, by proposing a public peer-to-peer electronic monetary system [7].

In 2013, Vitalik Buterin proposed the Ethereum platform, which went live in 2015. Ethereum widens the concept of transactions to represent arbitrary state changes in a ledger [8]. This allows modelling general purpose concepts programmatically on the blockchain [3]. Ethereum, in contrast to Bitcoin, is a blockchain technology that allows the embedding of code in the blockchain and execution of it within the network. The term “smart contract” refers to this ability. Most blockchain projects today do not create a new blockchain network, but rely on Ethereum, with its ability to execute smart contracts [9].

It is noteworthy that “Bitcoin” refers to the distributed network and technology, while “bitcoin” refers to the digital currency, as “Ethereum” refers to the technology and network, while “Ether” refers to the cryptocurrency.

## Basics of blockchain technologies

### Definition of blockchain and distributed ledger technology

**Fig. 1:**
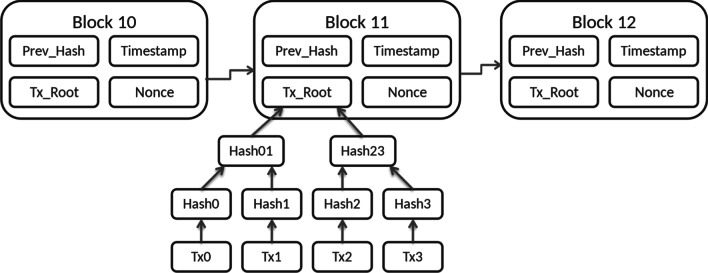
Transactions in a blockchain. Blocks, Nodes and Hash: Blockchain structure of Bitcoin: The data is stored in a continously growing list (chain) of records (blocks). Figure by: Matthäus Wander—Own work, CC BY-SA 3.0, https://commons.wikimedia.org/w/index.php?curid=26816920

Blockchain stores data in a distributed network relying on many nodes instead of one central control node. This network is called the distributed ledger. Data is stored in an immutable non-modifiable (i.e., write once) fashion, making illicit modifications of the data extremely difficult. Data can only be added to the blockchain, and blocks cannot be removed or modified once they have been written. The data stored is thus a continuously growing list of records (blocks), appended one to another (chain). “Blockchain is a type of distributed database that stores a permanent and tamper-proof ledger of transaction data.” [10] (Fig. 1).

The distributed ledger database is spread across several nodes as devices on a peer-to-peer network, where each node replicates and saves an identical copy of the ledger and updates itself independently. The primary advantage is the lack of a central authority or central server. When a ledger update happens, each node constructs the new transaction, and then the nodes vote by consensus algorithm on which copy is correct. Once a consensus has been determined, all the other nodes update themselves with the new, correct copy of the ledger. Security is accomplished through cryptographic keys and signatures [11–13]. Data quality is maintained by database replication and computational trust.

Blockchains are one form of distributed ledger technology: not all distributed ledgers employ a chain of blocks to provide a secure and valid distributed consensus. However, the structure of the blockchain, where data is grouped together and organized in blocks, with blocks linked to one another and secured using cryptography, makes it distinct from other kinds of distributed ledgers. Blockchain technology is therefore well-suited for recording events, managing records, processing transactions, tracing assets, and voting [12].

### Blockchain compared to conventional database technology

Unlike conventional database technology, blockchain is decentralized, traceable and immutable.

*Decentralization* refers to the processes of data verification, storage, maintenance, and transmission on blockchain, which are based on a distributed system structure. In this structure, the trust between distributed nodes is built through mathematical methods rather than the centralized organizations with a single node in conventional database technology.

*Traceability* means that all transactions on the blockchain are arranged in chronological order, a block being connected with two adjacent blocks by the cryptographic hash function. Inherently, every transaction is traceable by examining the block information linked by hash keys.

*Immutable* means that tampering with any transaction would result in different hash values (hash keys link the previous block and point to the next block) and would thus be detected by all the other nodes running precisely the same validation algorithm. As blockchain is a shareable public ledger stored on thousands of nodes that are continually in sync in real time, successful tampering would need to change over 51% of the ledgers stored in the network [14].

### Public vs. private blockchains

Blockchain implementations exist in public and private variants. A public blockchain is open to all, and everybody can access it without invitation. A private blockchain has restricted access which is granted ‘by invitation only’ and only approved participants, like medical professionals, have access to the blockchain. This difference can be compared to the difference between the open access internet and an access only with approval intranet. Public blockchains offer a lower throughput for reaching the consensus due to their complexity and wide distributed nature. Private blockchains achieve higher update ratios. By their nature, cryptocurrencies such as bitcoin want to attract a maximum of participants and are public blockchain networks, while many enterprise applications use private blockchains in order to control access to the data stored on the blockchain [4].

## Applications of blockchain in medical imaging

### Clinical applications

#### Contribution of different “authors”, including AI systems, to multipart reports

Radiology reports of complex studies frequently contain input from more than one expert, especially in unusual cases, or those involving multiple body systems, specialties (e.g., combined radiologist/cardiologist reporting of cardiac imaging) or modalities (e.g., combined nuclear medicine physicians/radiologist reporting on PET/MR). While a unitary report will usually be synthesized from available inputs, blockchain offers the possibility of identifying whose opinion or expertise has contributed to each element of the report, thereby facilitating more-direct consultation with the appropriate contributor in cases where further information is desired. Reports can be structured in different blocks without the need for an encrypted blockchain. Blockchain can provide the exact level of responsibility of each contributor in the evaluation of a medical imaging study, as individual contributions will remain individually signed and the order in which each analysis was done is preserved.

#### Document the use of AI algorithms towards the diagnosis

As with multiple human contributors to a report, radiology reports may contain a combination of radiologist and AI-generated content, such as structured report forms pre-populated by AI algorithms. This is likely to be an increasingly common form of hybrid reporting in the near future. Again, blockchain facilitates differentiation of the different report elements, and assignment of responsibility for inclusion of information directly to a human radiologist, or to a radiologist relying on assistance from AI, while identifying which version of which AI algorithm has contributed.

#### Sharing of clinical data, traceability of reports and reading of reports

Blockchain-based electronic medical records (EMR) have been under discussion for several years. These EMRs would automatically store in the blockchain information about who contributed which part of the EMR. Coupled with algorithms designed to show relevant data to the remote radiologist, these EMRs would enable the radiologist to see the relevant data for the cases s/he is working on, without having to browse through large parts of the EMR. This in turn could result in more efficient and better interpretation of radiology studies. In addition, if the radiologist gives follow-up recommendations or describes how incidental findings should be managed, these items can also be stored and validated on the blockchain. The blockchain also allows storage of identification of who effectively read which parts of the EMR. This could be used in the future to track the follow-up of incidental findings. All of these potential uses together would enhance the quality of patient care.

#### Personal health record control and control of image data sharing

The control of data sharing ensuring user control is relevant for many applications, and platforms supporting user-controlled data sharing have been described [15]. More specifically, blockchain technology may be used to place owners of data stored in EMRs in control of their medical data. Thus, patients are empowered to share sensitive records with institutions of their choice for improved healthcare [16]. Systems letting clinicians make requests for patient data and enabling patients to grant or revoke access have been described [17, 18]. In a similar way, blockchain could also empower patients to control the sharing of their image data [19, 20].

### Applications of blockchain in research

#### Clinical trials framework for biomarker derivation

Clinical trials are key in informing changes in clinical practice. Rigorous trial conduct and an audit trail with absolute transparency are mandatory elements for a successful and reliable trial. As imaging is frequently a cornerstone of identifying disease progression or regression, blockchain technology has the potential to introduce a tamper-proof mechanism for recording imaging data within clinical trials. This encompasses all stages of image manipulation, analysis and quantitative assessment. Imaging data in trials is often derived from pre-specified imaging protocols, and variations in these, or their inaccurate recording, have potential to alter the images and therefore the measured outputs. The temptation to manipulate images prior to measurement is removed by the implementation of blockchain, ensuring the integrity of the images and their measurements. This kind of audit is of particular value when images are being uploaded or downloaded between participating sites to perform multiple measurements. Any change to image settings prior to making measurements, which potentially will influence outcomes, would be date- and time-stamped by the blockchain system, so acknowledging when and by whom the changes were made. This would avoid inappropriate data manipulation and ensure traceability of any significant changes [21]. Any data corruption would be identified without the need for human interrogation of the data [22]. In an era where commercial outputs often hang on the validity of imaging measurements, a robust method of documenting measurement history is vital.

Specific areas that could exploit blockchain technology within an imaging trials portfolio include lesion segmentation and implementation of analysis algorithms. Segmentation is traditionally manual, but increasingly is becoming semi- or even fully-automated. Often the manual segmentation data sets annotated by experts are used to train an algorithm to autonomously achieve a similar result. Documentation of the training sets, and their adjustments in the context of experts and software would provide insights into the basis for the machine-learnt outputs and explain unexpected variations. Data groups for each block could be assigned by patient visit, or by groups of examinations at specific time-points within the trial.

Overwhelming evidence of bias exists when commercial or academic investigators analyze and report their own findings [23, 24]. Blockchain technology applied to imaging within clinical trials would deliver provenance-assured data sets to third parties for analysis. This would separate the hypothesis driving the trial from the expected results. A particular bonus would be in secondary research exploiting data sets from imaging biobanks, where the integrity of the raw data, especially when pooling data from multiple trials, could be assured.

#### Collection of data for scientific purposes, especially for the training of AI algorithms

One interesting application of blockchain is to document who has contributed to the data for the training of AI algorithms, including patients with their data, radiologists with annotations, and industrial partners with developing the AI algorithm. This annotation allows the distribution of financial rewards to different partners, which in turn might be a motivation for patients and radiologists to participate in the process. The use of blockchain technologies would also allow patients and radiologists to track usage of their data and thus be in better control of their files. It has been shown on experimental benchmark image datasets that the need for accuracy, privacy and fairness in collaborative deep learning can be effectively addressed by using a combination of distributed and federated deep learning and blockchain [25].

These properties of blockchain may also help to make data for AI training available in large quantities. To train supervised deep learning networks, one needs as much high quality data and annotations as possible. If the dataset used for training is not large enough, rare events will not be reliably detected, resulting in a selection bias which will affect the generalizability of the AI system. Since we usually lack direct insight into the internal operations of deep learning models, biases can be insidious and become dangerous [26]. A blockchain-based AI algorithm can not only learn from shared data from multiple institutions, but it also enables engineers to track and evaluate its learning by looking back or simply replaying the chain, giving more insight and greater human oversight of AI decision making [27]. This is indeed a key value of blockchain, akin to the tracing of products from producers to consumers. The annotation of the conditions in which a model was trained undoubtedly provides information on its quality.

A further benefit of blockchain could be its use in attribution of the source of material used for teaching and educational resources. Blockchain could ensure appropriate attribution of the source of contributions to educational material, potentially increasing willingness on the part of primary authors to share material, without loss of intellectual property credit.

#### Dynamic consenting

Dynamic consent is a new way to empower research partners and facilitate active participation in the research process. In the Dwarna project, it has been shown in the context of biobanking that the use of blockchain technology might give individuals access to information and control to determine how and where their biospecimens and data should be used [16], facilitating compliance with the right to erasure mandated by the European Union’s General Data Protection Regulation (GDPR) when using the blockchain model.

#### Empowering patients

The current way to store images in central databases and to transfer them often using physical media not only results in delays in patient pathways, but also exposes the data to tampering. Patel has developed a framework for cross-domain image sharing that uses a blockchain as a distributed data store to establish a ledger of radiological studies and patient-defined access permissions [20]. This framework allows secure and decentralized sharing of medical data. It also empowers patients by allowing them to effectively own their image data and to control healthcare provider access privileges [28].

### Using the blockchain for administrative purposes

#### Learning, tracking of learning

Blockchain may be used to document and keep track of learning achievements by maintaining digital hashes of learning accomplishments and managing access rights through the use of smart contracts on the blockchain [29]. Ocheja et al. described a blockchain of learning logs (BOLL) that enables learners to move their learning records from one institution to another in a secure and verifiable format [30]. Nasseem et al. analyze the potential use cases for blockchain deployment in medical education ecosystems, to improve the efficiency, security, functionality and effectiveness of existing infrastructures. They propose using blockchain to eliminate the problem of fraudulent academic accreditations [31].

#### Surveillance of medical devices

For the increasingly important and complex post-marketing surveillance (PMS) of medical devices, increasingly large amounts of data are being generated. It has been shown that a private data-permissioned blockchain with a proof-of-authority consensus mechanism could offer many advantages to the different stakeholders involved in the PMS process, such as providing support with new regulatory initiatives [32].

### New business models arising from the use of blockchain in radiology?

As of today, many AI applications have proven their potential to enhance the interpretation of medical imaging. One of the main obstacles in developing new AI algorithms with supervised deep learning is the lack of large numbers of high quality annotated images. While the motivation of AI companies is to generate benefits by selling the algorithms, achieving motivation of patients to contribute their medical images, and of radiologists to do the extra work of image annotation, is less straightforward. Using blockchain technologies to track who has contributed might help to overcome these obstacles by granting tokens or rewards to those who have contributed.

Blockchain can also be used for consensus annotation, where annotations are approved only when there is consensus about the annotation among multiple participants, rewarding those in consensus and penalizing those not in consensus [3]. Blockchain can also help to create large open databases with data contributions from many different sources. Blockchain also facilitates access protection of personal health data and protection of data integrity.

While most patients would agree to share their health data if it is used for the general good, patient agreement might be less forthcoming when profits are made from their data by pharmaceutical or imaging companies. It would be motivating and fair if there were a financial incentive for patients to contribute their data. The same applies for radiologists and the annotation of data; if profit is made from the annotations, it would be fair to share some of the profits with those who have done the annotation work. Blockchain allows the users, both patients and radiologists, to remain anonymous and to keep control of their data or annotations. They can decide to contribute them for free, or to be rewarded for their use [33].

By creating a win–win situation for all parties involved in the creation of deep learning AI algorithms, including patients giving their data, radiologists annotating them, and companies training and implementing the algorithms, blockchain could help to overcome the bottleneck of the availability of image data with high quality annotation (Table 2).

**Table 2:** Potential use cases of blockchain in radiology

Domain	Application	Proposed data blocks
Clinical	Personal health record control Control of image sharing (patient-driven ownership) Control of image data integrity Tracking incidental findings	Multimodality data from individual patient visits, data from multiple timepoints through a single course of treatment
Administrative	Supply chain tracking and management Imaging equipment maintenance and inspection record keeping Societal voting	
Research and machine learning	Data sharing Keeping track of clinical trials Machine Learning: training and AI execution	All data from individual trials, data analyzed by individual investigators of using specific algorithms

## Summary

Blockchain is an extremely powerful technology facilitating storage of provenance data in an immutable way, providing traceability of all modifications to stored data. Blockchain provides trustful information on how, who, when and where data was generated. It has numerous applications in medicine and radiology. Blockchain has the potential to empower both patients and radiologists, by allowing them to keep control of the use of their data and / or annotations of data. It is also well suited to storing patient data for clinical use. The radiology community should engage and collaborate in the development and implementation of blockchain technologies in research and patient care.

## Data Availability

Not applicable.

## Abbreviations

AI: Artificial intelligence
DLT: Distributed ledger technologies
EMR: Electronic medical records
GDPR: General data protection regulation
PMS: Post-marketing surveillance
